# Data-Driven Virus Discovery with Virushunter and Virusgatherer

**DOI:** 10.1093/nargab/lqag120

**Published:** 2026-10-13

**Authors:** Liubov Chuprikova, Li Chuin Chong, Sergej Ruff, Stefan Seitz, Chris Lauber

**Affiliations:** Division of Virus-Associated Carcinogenesis (D430), German Cancer Research Center (DKFZ), 69120 Heidelberg, Germany; Medical Faculty Heidelberg, Department of Infectious Diseases, Molecular Virology, Center for Integrative Infectious Disease Research, Heidelberg University, 69117 Heidelberg, Germany; Institute for Experimental Virology, TWINCORE Centre for Experimental and Clinical Infection Research, a joint venture between the Hannover Medical School (MHH) and the Helmholtz Centre for Infection Research (HZI), 30625 Hannover, Germany; Institute for Experimental Virology, TWINCORE Centre for Experimental and Clinical Infection Research, a joint venture between the Hannover Medical School (MHH) and the Helmholtz Centre for Infection Research (HZI), 30625 Hannover, Germany; Institute of Animal Genomics, University of Veterinary Medicine Hannover, 30559 Hannover, Germany; Division of Virus-Associated Carcinogenesis (D430), German Cancer Research Center (DKFZ), 69120 Heidelberg, Germany; Medical Faculty Heidelberg, Department of Infectious Diseases, Molecular Virology, Center for Integrative Infectious Disease Research, Heidelberg University, 69117 Heidelberg, Germany; Institute for Experimental Virology, TWINCORE Centre for Experimental and Clinical Infection Research, a joint venture between the Hannover Medical School (MHH) and the Helmholtz Centre for Infection Research (HZI), 30625 Hannover, Germany; Cluster of Excellence 2155 RESIST, Hannover Medical School, 30625 Hannover, Germany

## Abstract

We present Virushunter and Virusgatherer, a bipartite computational workflow for discovering viral genomes from unprocessed next-generation sequencing data. Virushunter conducts a profile Hidden Markov Model-based sequence homology search for viral hallmark protein-coding sequence reads to construct microcontigs, which are used as seeds for targeted viral genome assembly by Virusgatherer. We showcase the application of Virushunter and Virusgatherer through an RNA virus-wide screen in 3348 bat transcriptome sequencing experiments from the Sequence Read Archive. Many of the discovered viruses are likely prototypes of new putative virus species, including a novel alphacoronavirus identified in a South American velvety free-tailed bat and a novel rhabdovirus in a European barbastelle bat. The tools are implemented as a reproducible Snakemake pipeline, which is available at https://github.com/lauberlab/VirusHunterGatherer.

## Introduction

Virus discovery has undergone significant advancements in recent years, driven primarily by the massive increase in next-generatiion sequencing (NGS) data and the parallel development of sophisticated data analysis methods. While traditional virus discovery approaches often relied on sample collection, experimental cultivation, and isolation techniques, Data-Driven Virus Discovery (DDVD) [1] leverages vast amounts of publicly accessible NGS data, allowing unprecedented exploration of viral diversity in high throughput [2–7]. DDVD enables researchers to uncover a broad spectrum of viruses, including those with low sequence similarity to known reference viruses [2, 7, 8]. This advancement has opened new avenues for understanding viral evolution, ecology, and virus-host interactions.

Here, we present Virushunter and Virusgatherer, a two-stage computational pipeline that implements a DDVD approach designed to discover both known and divergent novel viruses from primary sequencing data in a high-throughput manner. The first stage, Virushunter, conducts a profile Hidden Markov Model (pHMM)-based homology search in the raw read data to identify the presence of sequences coding for viral hallmark proteins. Detected viral sequencing reads are assembled to microcontigs that serve as seeds for Virusgatherer, the second stage of the workflow, which performs targeted assembly of the viral genome sequence. Both user-provided and public sequencing data, e.g. from the Sequence Read Archive (SRA) of the National Center for Biotechnology Information (NCBI), can be used as input for Virushunter and Virusgatherer, and the results are summarized in the form of hit tables and can be visualized using the accompanying R package Virusparies [9]. We demonstrate the pipeline through an RNA virus-wide screen in 3348 publicly available bat transcriptome sequencing experiments from the SRA.

## Materials and methods

### Pipeline design and implementation

The Virushunter & Virusgatherer DDVD pipeline has been used in previous studies [2, 6–8, 10] and is summarized in Fig. 1. The main pipeline stages include data acquisition, virus detection through Virushunter, and viral genome assembly through Virusgatherer. Results from Virushunter and Virusgatherer are provided as tab-separated hit tables, which can be visualized using the accompanying R package Virusparies, and as FASTA files containing assembled viral genome sequences. The pipeline steps are implemented as separate rules in the Snakemake (v9.17.1) workflow management system [11]. All pipeline parameters can be set by the user through the YAML configuration file. The pipeline is optimized for Linux operating systems and depends on several publicly available software tools and reference sequence databases for both hosts and viruses, which can be retrieved from NCBI (see below).

**Figure 1.**
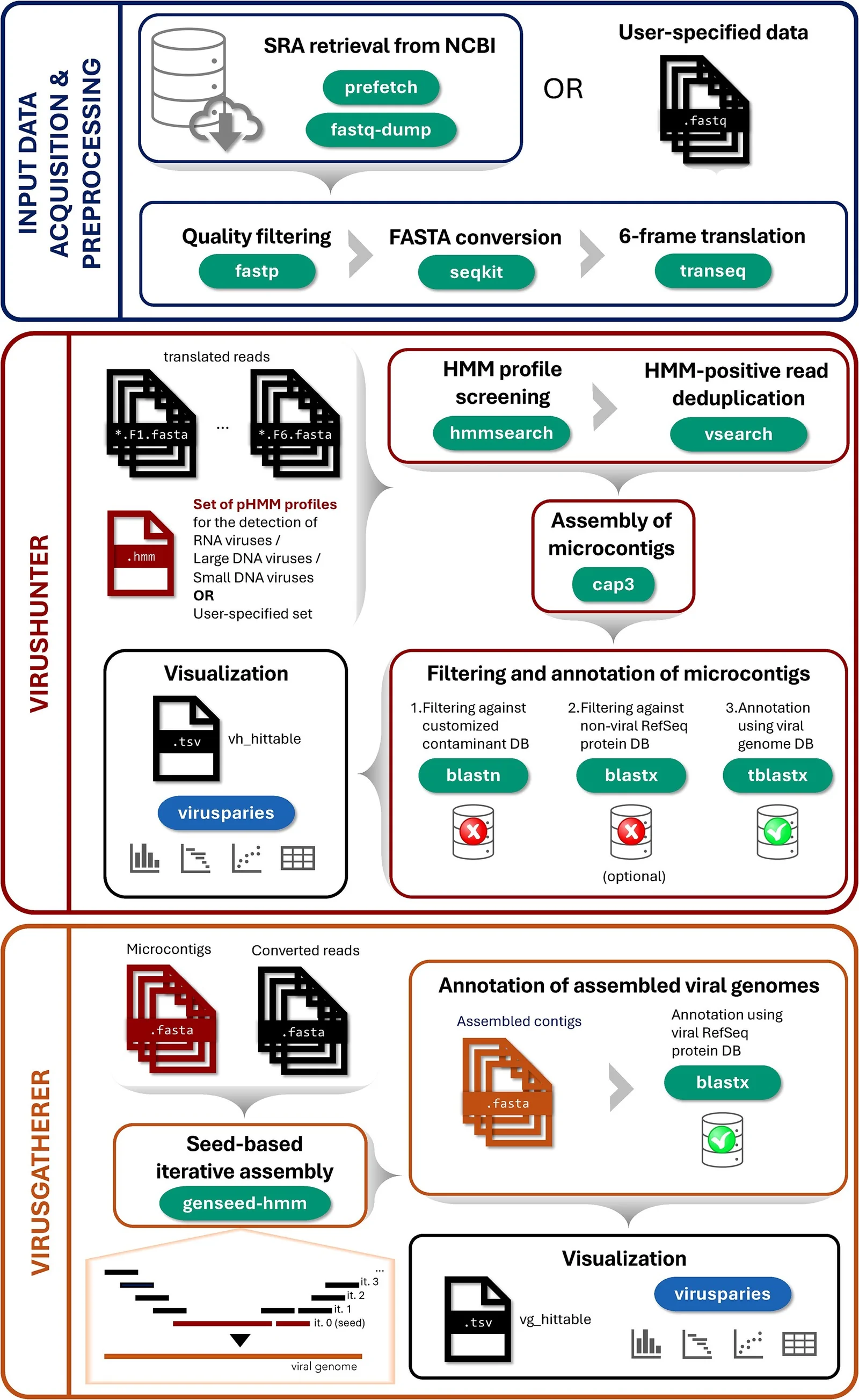
Overview of the Virushunter and Virusgatherer pipeline. The pipeline comprises three main stages: (i) input data acquisition and preprocessing from public (NCBI SRA) or user-provided sequencing data, including quality filtering, conversion to FASTA and translation into six reading frames; (ii) virus detection with Virushunter, where *in silico* translated reads are screened against viral HMM profiles, deduplicated, assembled into microcontigs that serve as seeds in the subsequent assembly step, and filtered/annotated against reference databases; and (iii) viral genome reconstruction with Virusgatherer, which performs seed-based iterative assembly and final annotation of assembled viral genomes. Results are provided as FASTA files and tab-separated hit tables that can be visualized using the accompanying Virusparies R package.

The pipeline and installation instructions are available at https://github.com/lauberlab/VirusHunterGatherer. We provide a vhvg.yml file to install dependencies using the Conda package manager.

### Input data acquisition and preprocessing

Given a list of SRA accession numbers, the pipeline downloads sequencing data from NCBI’s SRA database using prefetch and fastq-dump from the SRA Toolkit (v3.2.1) (Fig. 1, top panel) [12, 13]. This step is bypassed if the user provides sequencing data in FASTQ format as input. For paired reads, a single interleaved FASTQ file should be generated for each sample. The preprocessing step involves trimming adapter sequences and low-quality bases using fastp (v1.1.0) with default parameters [14]. The trimmed reads are then converted from FASTQ to FASTA format using seqkit (v2.13.0) [15] and *in silico* translated into six reading frames using transeq from the EMBOSS suite (v6.6.0) [16].

### Detecting sequencing reads of viral origin with Virushunter

Virushunter utilizes pHMMs, which were typically constructed at the virus family level or higher taxonomic ranks, to screen the raw (unassembled) sequencing data for the presence of reads that share significant sequence similarity to viral marker genes (Fig. 1, middle panel). Ideally, these marker genes have no close homologs in cellular genomes to avoid false-positive hits. We provide three sets of pHMMs built on multiple sequence alignments of distinct group-specific key proteins that cover a broad range of (i) RNA viruses, (ii) DNA viruses with small genomes, and (iii) DNA viruses with large genomes (Supplementary text, Supplementary Table S1). Moreover, users can employ a custom set of pHMMs to screen for any additional virus families (or even mobile genetic elements or cellular genes) of their choice.

The sequence homology search is performed by hmmsearch from the HMMER suite (v3.4) [17] against the 6-frame translated reads. Reads with an E-value smaller than 10 are deduplicated using vsearch (v2.30.5) with “–notrunclabels” but otherwise default parameters [18] and assembled with CAP3 (Bioconda package v10.2011) with parameter “-h 75 -o 20” into microcontigs (can be as large as the employed marker gene) [19]. Deduplication of the identified viral reads was performed to reduce redundancy and accelerate the subsequent assembly into microcontigs, reducing the number of sequences subjected to downstream filtering and annotation. The resulting microcontigs are filtered by comparing them against a custom database of common contaminants and, optionally, a nonviral reference protein database to remove false-positive hits. The database used in the latter step (such as those provided by NCBI) has to be carefully considered to rule out contamination with unrecognized viral proteins, which otherwise would result in removal of true viral hits from the Virushunter output. The remaining microcontigs are then checked against the NCBI viral reference genome database, applying a moderately permissive E-value cutoff of 1 to retain potentially divergent viral sequences as seeds for the subsequent iterative assembly by Virusgatherer.

### Assembling viral genome sequences with Virusgatherer

Virusgatherer performs a targeted assembly of viral sequences using Genseed-HMM (v1.0.6) [20] with the viral microcontigs identified by Virushunter as initial seeds and parameters “-ext_seed_size 30 -blastn_parameters “-evalue 0.001 -perc_identity 85 -word_size 7 -dust no -soft_masking false“ -tblastn_parameters ”-max_target_seqs 50 000“ -clean no -duplicate_headers no -max_contig_length 50 000 -max_number_rounds 100 -mapping no -align_threshold 50 -use_qual no”; in case of using CAP3 as the assembler, we additionally set “ -assembler_parameters ”-o 20 -h 99 -p 85″ .” (Fig. 1, bottom panel). During iterative contig extension, the ends of the current contig are used as queries to search in blastn mode (BLAST + v2.17.0) for additional reads or read pairs aligning to the contig ends. To provisionally annotate the assembled contigs with taxonomic information, they are compared against NCBI’s database of reference viral proteins (viral RefSeq) via blastx with the default E-value cutoff of 10, allowing even divergent contigs to be assigned to their closest reference sequence.

Genseed-HMM supports multiple internal assembly programs; we recommend using CAP3 [19], with Newbler being an alternative [21]. Conducting several analyses with different assemblers and combining the results can be a useful strategy in some instances. We distribute a modified version of the original Genseed-HMM code. First, we added a deduplication step after the search for additional reads in each iteration, which significantly decreases CAP3 assembly time for high copy-number viruses. Second, we modified the method for retrieving FASTQ reads due to incompatibilities with recent BLAST versions.

Virusgatherer contigs were clustered using MMSeqs2 (version 15–6f452) with parameters “-c 0.5 –cluster-mode 2 –min-seq-id 0.9,” [22] which is not part of the Virusgatherer workflow.

### Reporting and visualizing Virushunter and Virusgatherer results

Both Virushunter and Virusgatherer generate tab-separated hit tables that summarize the results. A Virushunter hit table provides an initial overview of the viral genomes expected to be present in the sequenced samples, while a Virusgatherer hit table summarizes the viral genome sequences that were assembled from the data. Both hit tables share columns such as the SRA run accession, sample, and study identifiers, along with the host taxon name and taxonomic identifier, all of which are automatically retrieved from the SRA metadata. The Virushunter and Virusgatherer hit tables also provide information about the best match to NCBI’s reference viruses (including subject name, subject taxonomy information, E value, percent amino acid identity). Unique to a Virushunter hit table are columns detailing the number of identified reads and (intermediate) microcontigs per input dataset, as well as the best-matching pHMM profile (name and E-value). Columns unique to a Virusgatherer hit table are viral contig name and length.

To facilitate inspection and visualization of Virushunter and Virusgatherer results, we provide the accompanying R package ‘Virusparies’ available at CRAN (https://cran.r-project.org/web/packages/Virusparies). Virusparies offers functions to subset and filter hit tables, calculate summary statistics, and generate plots and graphical tables.

## Results

We demonstrate the application of the Virushunter and Virusgatherer pipeline through an analysis of 3348 publicly available transcriptome experiments (as of August 2025) of bat origin obtained from the SRA database (Supplementary Table S2). This dataset encompasses sequencing data from 154 species, 79 genera, and 17 families of bats (Supplementary Table S3). The SRA run accession list was obtained by querying the SRA website with the taxonomic identifier of the order Chiroptera (taxid 9397) and limiting the result to transcriptomic datasets (source RNA). Virushunter and Virusgatherer showed an average processing time of about 90 + 110 minutes per GB of data, with a sensitivity of 96% and a specificity of 87% in a validation analysis of *in silico* generated sequencing datasets simulating virus-positive and -negative experiments (Supplementary text, Supplementary Fig. S1); contig assembly completeness varied considerably from 1% to 100% depending on simulated coverage depth, with an overall average of 53.5% and the strongest increase observed from depth 10 to 20 (Supplementary text, Supplementary Fig. S2).

### Identification of putative viral sequences using Virushunter

We screened the bat transcriptome experiments for the presence of RNA virus sequences by utilizing a comprehensive collection of 31 RNA virus pHMMs in the Virushunter stage (see Supplementary text for details). The pHMMs were constructed at the family level or higher taxonomic ranks and were mostly based on multiple sequence alignments of the hallmark protein of RNA viruses, the RNA-dependent RNA polymerase (RdRp) (Supplementary Table S1). Virushunter gave hits in 1285 (∼38.4%) of the 3348 analyzed datasets, of which 1229 produced significant hits (E-value ≤ 1e−5) in a protein BLAST (tblastx) comparison against RNA virus reference sequences. In total, 4144 unique microcontig hits against different RNA viral reference sequences were obtained, corresponding to an average of 3.2 hits per sequencing experiment (Supplementary Table S4). Among the hits were reference viruses from 74 different RNA virus families but also false-positives from four DNA virus families (Supplementary Fig. S3). Figure 2A, produced using the accompanying R package ‘Virusparies', illustrates the 20 virus families that ranked top with respect to the number of SRA datasets that were hit. The number of detected sequencing reads of putative viral origin, which is one of the factors for success of viral genome assembly in the subsequent Virusgatherer stage (see below), varied considerably across the different RNA virus families (Fig. 2B). For instance, although the number of datasets with *Bornaviridae* sequences was the largest (*n* = 170), only a relatively low number of reads were detected (*r* = 579 in total), suggesting that many of these hits may come from endogenized bornavirus genomes, which are known to be frequent in vertebrates [23]. In contrast, *Spinareoviridae* sequences were found in only 43 SRA datasets but showed by far the largest number of detected reads (*r* = 49 201) (Fig. 2B), assembling into 91 microcontigs (Fig. 2C), suggesting high copy numbers of viral genomes in the positive sequencing experiments, which would be expected for actively replicating RNA viruses. The E-values in the filtering BLAST analysis against viral reference genomes ranged from 1e−5 to 1e−173 (Fig. 2D) where higher E-values often indicate hits against viruses that diverged considerably from the most closely related known reference virus.

**Figure 2.**
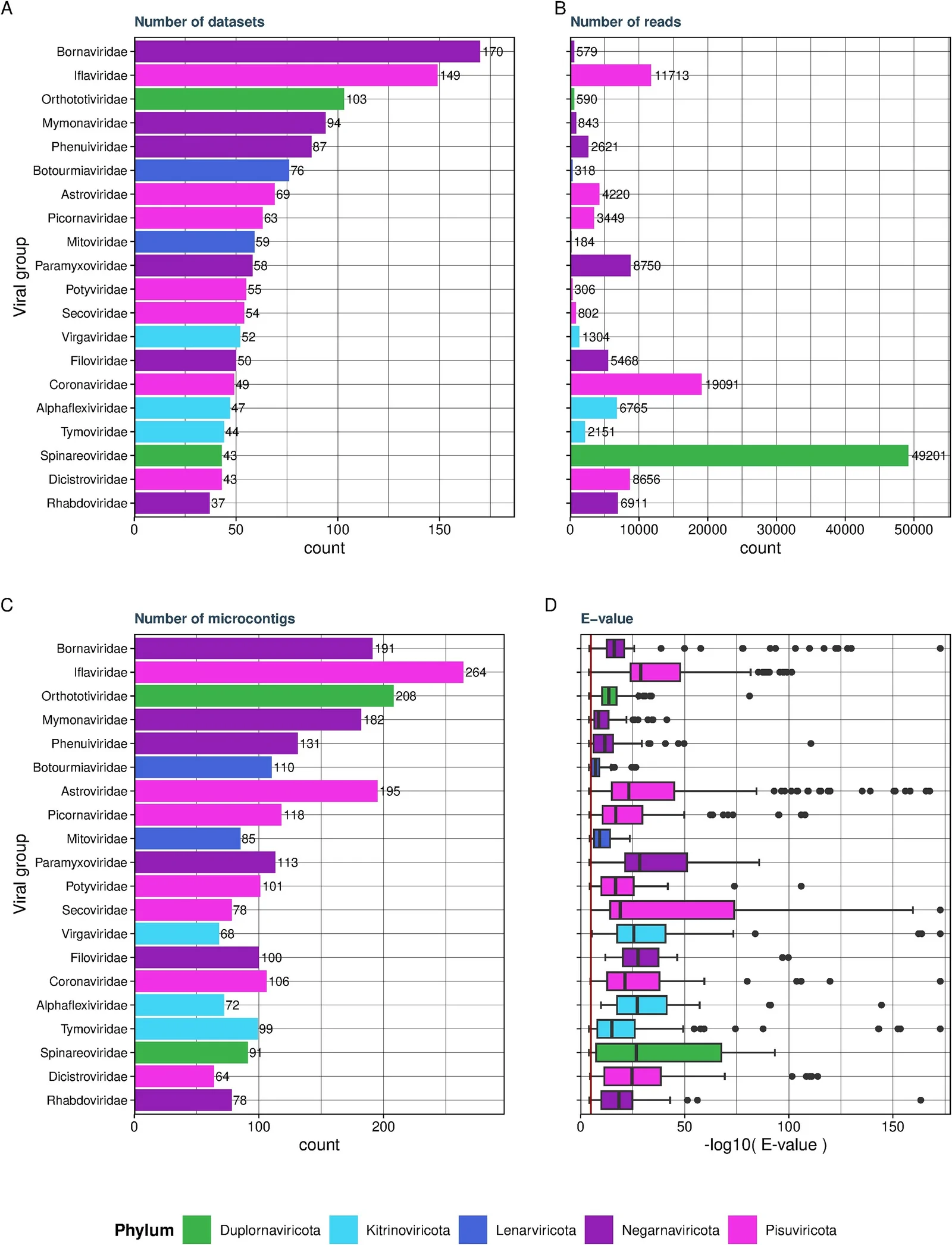
Virushunter results. Results are shown for the 20 RNA virus families with the largest number of SRA datasets in which one or several hits were obtained. Virus families are colored by RNA virus phylum. (**A**) Number of SRA datasets. (**B**) Number of sequencing reads summed over all datasets. (**C**) Number of microcontigs summed over all datasets. (**D**) Distribution of E-values from protein-based BLAST (tblastx) comparison of microcontigs against reference genomes from viral RefSeq. Numbers in panels (A–C) indicate absolute counts. All graphs generated using the accompanying R package Virusparies.

Virushunter detects sequence homology to viral marker genes, which constitute a small part of the viral genome and therefore provides a first rough overview of the number of discovered putative viruses and their closest known relatives. Viral genome assembly through the Virusgatherer stage is necessary to validate the presence and determine the identity of the viral genome sequences.

### Targeted viral genome sequence assembly using Virusgatherer

Virusgatherer assembled 4329 contigs ranging from 57 to 27 392 nt in length (Supplementary Table S5, Supplementary Fig. S4). To account for potential sequence redundancy, we clustered the contigs at 90% nucleotide sequence identity, resulting in 3074 contigs, resembling species-like operational taxonomic units (OTUs) with an average length of 497 nt, and 215 (7.0%) and 68 (2.2%) of the contigs being longer than 1000 and 5000 nt, respectively. The resulting viral contigs were assigned to 80 RNA virus families with E-values in the range of 1e−5 to 1e−180 or remained unclassified at the family rank and showed protein sequence identities to viral reference sequences between 22% and 100% with an average of 69.6%. We cannot exclude that some of the contigs with high E-values represent false-positive hits. Of the 3074 species-like OTUs, 2441 (79.4%), 1583 (51.5%), and 534 (17.4%) showed identities to viral RefSeq proteins lower than 90%, 70%, and 50%, respectively, while 156 (72.6%) of the 215 OTUs with contigs longer than 1000 nt showed sequence identities lower than 90% (Supplementary Table S5, Supplementary Fig. S4). Among the 20 most abundant RNA virus families were several families known to infect mammals, such as the families *Astroviridae, Paramyxoviridae, Picornaviridae, Coronaviridae, Phenuiviridae*, and *Filoviridae*, but also families whose members are thought to infect invertebrates and even fungi and plants (Fig. 3). Thirty-two of viral contigs were 5000 nt or longer and showed sequence identities to the closest RefSeq representative of < 80% (Supplementary Figure S5).

**Figure 3.**
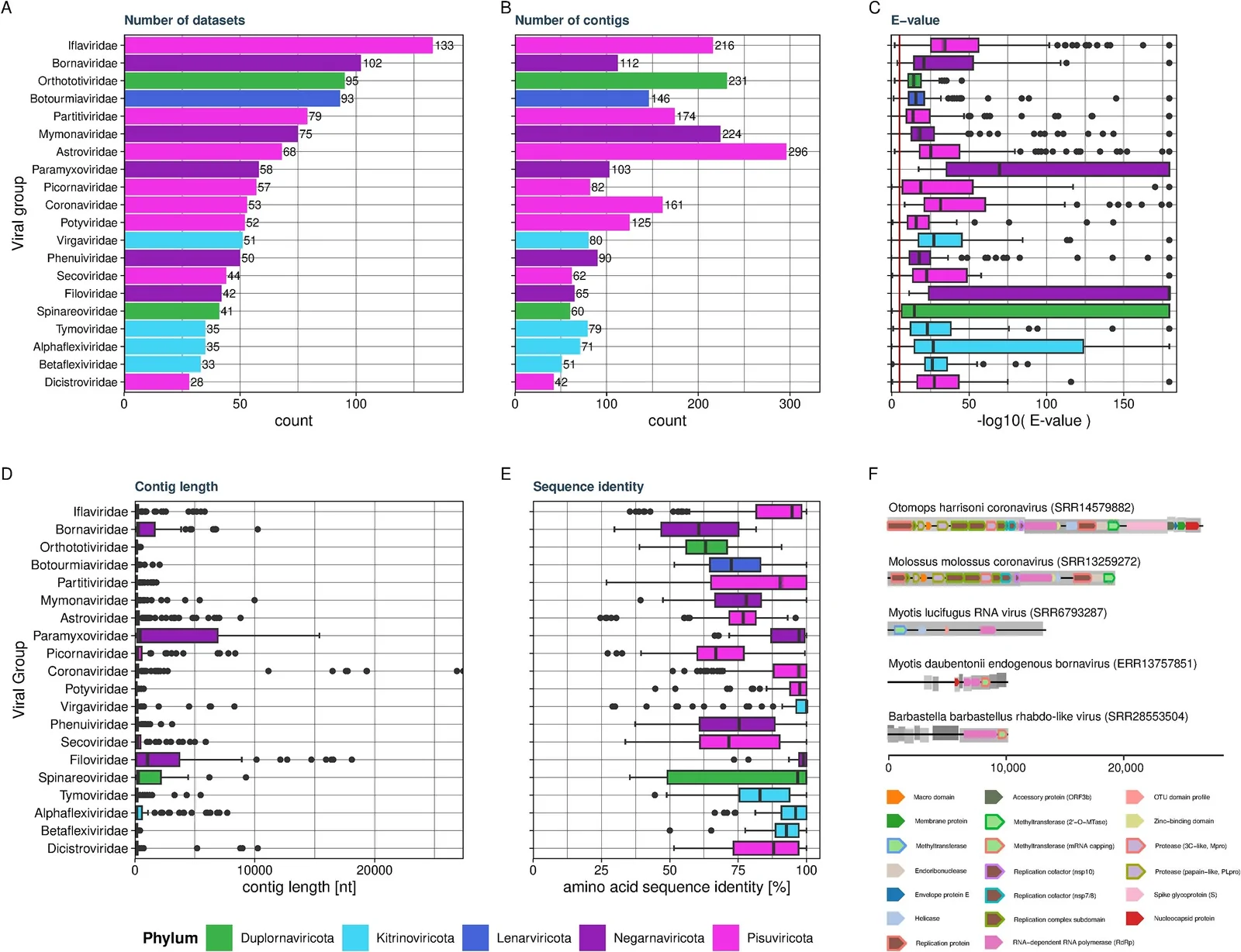
Virusgatherer results. Results are shown for the 20 RNA virus families with the largest number of SRA datasets in which viral contigs were assembled. Virus families are colored by RNA virus phylum. (**A**) Number of SRA datasets. (**B**) Number of assembled contigs summed over all datasets. (**C**) Distribution of E-values from protein-based BLAST (blastx) comparison of contigs against reference proteins from viral RefSeq. (**D**) Distribution of contig lengths. (**E**) Distribution of percent amino acid sequence identities from protein-based BLAST (blastx) comparison of contigs against reference proteins from viral RefSeq. (**F**) Genome maps of five selected viral contigs assembled by Virusgatherer. The contig from SRR13259272 is partial and does not cover the complete protein-coding region of the viral genome. Predicted open reading frames (ORFs) of at least 300 nt are shown as rectangles with different shadings of gray. Protein domains predicted in an InterProScan analysis are shown as colored shapes within ORFs; see color legend at the bottom. Viruses are named by the bat species in which they were discovered and the most closely related virus reference taxon; corresponding SRA identifiers are in brackets. The scale bar is in nucleotides.

### Discovery of relatives of important human pathogens

Among the discovered RNA viruses were known human pathogens, including Zaire ebolavirus, Marburgvirus, and Severe acute respiratory syndrome coronavirus 2 (SARS-CoV-2) (for instance from project PRJNA1205767 studying the SARS-CoV-2 furin cleavage site in bat cells). We also found viruses closely related to previously reported bat viruses, such as Bat hepacivirus (contig length of 9066 nt; amino acid sequence identity of 99.97% to accession DAD52839.1), Bat hepatitis E virus (6654 nt; 93.73% to XMO98526.1), or Bat morbillivirus (9531 nt; 99.89% to DBA64462.1) (Supplementary Table S5, Supplementary Fig. S4).

Moreover, we identified a large number of viral genomes with amino acid sequence identities to viral RefSeq entries of well below 90% (Fig. 3E and F, Supplementary Fig. S4E). Among them was a partial genome sequence (19 350 nt) covering the ORF1a/b region of a novel coronavirus identified in an intestine sample of a velvety free-tailed bat (species ‘Molossus molossus’) from Peru (SRA dataset SRR13259272). This novel coronavirus showed an amino acid sequence identity of ∼70.63% to a bat alphacoronavirus (Bat Coronavirus EsJX20, accession WCC63170.1), suggesting that it is a prototype of a new virus species within the genus *Alphacoronavirus*. We also identified a coding-complete genome (10 242 nt) of a novel rhabdo-like virus in a blood sample of a barbastelle bat (species ‘Barbastella barbastellus’) from the United Kingdom (SRA dataset SRR28553504) that showed an amino acid sequence identity of only ∼46.65% to an unclassified rhabdovirus (Agrotis ipsilon virus, accession QKY64636.1) (Fig. 3F, Supplementary Table S5). See Supplementary text for more details about the viral genomes shown in Fig. 3F.

## Discussion

The Virushunter and Virusgatherer DDVD pipeline enables the large-scale screening of raw sequencing data for the presence of viral marker genes and the subsequent reconstruction of respective viral genome sequences, as demonstrated in previous studies reporting members of novel virus species, genera, or entire families, including nackedna—and hepadnaviruses, ambiviruses, as well as corona—and other nidoviruses [2, 6–8]. It also enables the discovery of new RNA virus orders [24]. Although we run the presented analysis in a high-performance computing environment with 12 to 128 CPUs and a memory of 2 to 4 GB per CPU per SRA experiment, it can be deployed on any modern UNIX-based system.

The pipeline implements a sequence homology search approach based on pHMMs that has been shown to be more sensitive than sequence-versus-sequence search methods such as BLAST [17]. Therefore, it allows for detecting divergent viruses [2, 6–8] that share amino acid sequence identity with reference viruses in the “twilight zone” of only 20%–30% [25], similar to other pHMM-based approaches [5, 26–30]. To account for the trade-off between sensitivity and specificity, we recommend downstream analyses of the discovered contigs, examining genomic organization, encoded proteins, and potential premature stop codons or frameshift mutations, to discriminate endogenous viral elements from extant viruses and to identify any remaining false-positive hits against host sequences.

In the case of RNA viruses, as analyzed here, the provided pHMMs primarily target the RdRp. Consequently, only the RdRp-encoding segment of a segmented viral genome will be identified. This shortcoming can be addressed by extending the standard pHMM set with segment-specific pHMMs or using a custom set of pHMMs tailored to particular virus groups.

The degree of “novelty” of an identified virus is based on shared sequence identity to reference viruses and inherently depends on the composition of the viral reference genome database employed in Virushunter and Virusgatherer. We therefore suggest regularly updating the locally installed reference sequence databases to the most recent NCBI versions to ensure accurate annotation and novelty assessment.

A key distinction from most other virus identification tools is the targeted, seed-based genome assembly approach implemented in Virusgatherer, which is applied *after* virus identification by Virushunter based on hits in the raw-read data. This strategy has two advantages compared to most standard approaches for computational virus discovery: First, it avoids assembling virus-negative datasets, and second, it is usually faster than untargeted assembly for virus-positive datasets because it excludes the large background of nonviral sequences. Moreover, the targeted approach does not make strong assumptions about the data, such as mean coverage of contigs, and, in theory, may work with a coverage depth approaching 1. Nevertheless, the success of assembling coding-complete (RNA) viral genomes depends on several factors, including the genetic heterogeneity of the sequenced viral population and the amount of viral genetic material present in the sequenced sample. Genomic regions of high heterogeneity can lead to premature termination of the seed-based, iterative assembly process. Standard, untargeted *de novo* assembly could help in some of these cases and may produce longer contigs, at the cost of expected larger resource consumption. The issue of insufficient amounts of viral sequencing reads can only be solved by resequencing the sample [6] and is typically due to the original SRA study not being dedicated to virus discovery. Notwithstanding that, even partial viral genomes provide important insights into previously undescribed viral genetic diversity and viral host range.

Virushunter and Virusgatherer offer approximate taxonomic assignments of the discovered viral genomes to the most closely related viral taxa recognized by the International Committee on Taxonomy of Viruses, but do not provide an accurate taxonomic classification. The clustering based on sequence identity thresholds, as conducted here, which is not part of the Virushunter and Virusgatherer workflow, offers a coarse estimation of the number of viral OTUs. We suggest employing coherent approaches and dedicated tools for the nontrivial and often family-specific task of virus taxonomy [31, 32].

Virushunter and Virusgatherer constitute a highly efficient computational pipeline originally dedicated to virus discovery in unprocessed NGS data. The pipeline can be easily updated and customized for this purpose or be repurposed for other, unrelated tasks, respectively, by adding new viral or nonviral pHMMs.

## Supplementary Material

lqag120_Supplemental_Files

## Data Availability

The Virushunter and Virusgatherer pipeline is available from GitHub at https://github.com/lauberlab/VirusHunterGatherer and FigShare at https://doi.org/10.6084/m9.figshare.33516694. Assembled sequence data accompanying the study have been uploaded to FigShare https://doi.org/10.6084/m9.figshare.31149460.
