# Supplementary material for: Data-Driven Virus Discovery with Virushunter and Virusgatherer
Source: NAR Genom Bioinform. 2026 Oct 13;8(4):lqag120. doi: 10.1093/nargab/lqag120 (PMC13659003; doi:10.1093/nargab/lqag120)
Supplement: Supplementary file 1 [file lqag120_supplemental_files.zip › SI_vhvg_manus_v06.pdf]

Supporting material for
Data-Driven Virus Discovery with Virushunter and Virusgatherer

Liubov Chuprikova*, Li Chuin Chong*, Sergej Ruff*, Stefan Seitz+, Chris Lauber+

* joint first author
+ joint last author

Correspondence to
Chris Lauber: chris.lauber@twincore.de
Stefan Seitz: s.seitz@dkfz-heidelberg.de

This PDF file includes:

Supplementary text section:
- Generation of multiple sequence alignments and profile HMMs
- Performance measures of Virushunter and Virusgatherer
- Reference databases
- Extended results

Supplementary figures S1 to S5
Additional table 1
SI references

Generation of multiple sequence alignments and profile HMMs

Multiple sequence alignments

Multiple sequence alignments (MSAs) of RdRps from different RNA virus families were obtained from (1). MSAs of major capsid proteins (MCPs) of 74 nucleocytoplasmic large DNA virus (NCLDV, phylum *Nucleocytoviricota*) lineages were obtained from (2). In addition, we compiled custom MSAs for virus families not covered by these published MSAs. The included groups of RNA and DNA virus taxa are listed in **Supplementary Table S1**. The custom MSAs were created by downloading available marker protein sequences, such as RdRp sequences in the case of RNA viruses, from Genbank, aligning them using MAFFT with default parameters and manually curating the resulting MSA. Information on marker proteins is given in the MSA names. For each MSA, we manually trimmed MSA termini. We also trimmed insertions that were exclusive to individual sequences. All MSAs have been uploaded to FigShare (see link in main text).

Profile HMMs

The MSAs were used to construct the following three sets of profile HMMs that accompany the Virushunter and Virusgatherer package: (i) 30 RdRp HMMs plus one for the Delta antigen of family *Kolmioviridae* covering the full spectrum of known RNA virus families (see also below), (ii) 12 HMMs of proteins diagnostic for DNA viruses with small genomes that include circular Rep-encoding single-stranded (CRESS) DNA viruses (3), hepadna- and nackednaviruses (4), anelloviruses, parvoviruses, papillomaviruses, polyomaviruses, and (iii) 88 HMMs of proteins diagnostic for DNA viruses with large DNA genomes that include NCLDVs, herpes- and adenoviruses.

Custom HMMs

The user can choose to use a custom set of profile HMMs. These should be constructed using hmmbuild from the HMMER3 package. The user needs to adjust the corresponding entries in the config.yaml configuration file of the pipeline. For more details, see the documentation at the Virushunter and Virusgatherer GitHub repository.

Performance measures of Virushunter and Virusgatherer

Generation of in silico sequencing datasets

To validate the accuracy of Virushunter and Virusgatherer we assessed their true-positive and true-negative rates by analyzing *in silico* generated sequencing datasets with and without known RdRp-encoding sequence content. Each generated dataset was composed of (i) a background sequence content derived from eukaryotic reference mRNA sequences and (ii) viral content derived from either RdRp-encoding or non-RdRp-encoding RNA virus sequences. No other sequences were included.

<u>Host background</u>: We downloaded all eukaryotic mRNA sequences of organism groups "vertebrate_mammalian", "vertebrate_other", "invertebrate", "plant", and "fungi" from NCBI RefSeq (as of 12th March 2026) and kept only those annotated as “known protein-coding” (accessions starting with ‘NM_’). These were 357,411 sequences in total. The original (host) organisms from which these background sequences originated are not relevant to this validation analysis, assuming that all included eukaryotic mRNAs encode proteins that are not evolutionarily related to viral RdRps. For each eukaryotic mRNA sequence we generated on average 1000 sequencing read pairs using InSilicoSeq (ISS) in basic mode (with default parameters) (5). This was achieved by using the full pool of eukaryotic mRNA sequences as input for ISS and requesting 1000 times the total number of analyzed sequences, resulting in varying read coverage depth per mRNA depending on mRNA sequence length. The ISS simulation in basic mode generated paired reads of length 125 nt without modeling sequencing mistakes. Finally, for each generated *in silico* dataset

(see below), we randomly selected one of the five eukaryotic groups. From this group we randomly selected 1000 mRNA sequence identifiers and extracted all ISS-generated read pairs for them.

RNA viral content: We downloaded all available NCBI RefSeq nucleotide sequences of RNA viruses (kingdom *Orthornavirae*, taxonomy identifier: 2732396). This included genome sequences of unsegmented and segmented viruses from a total of 119 virus families and 27 virus orders (note that this was prior to a major ICTV update in 2026). We excluded segmented viruses for which only a single segment was in the database. The total number of remaining reference sequences was 7708 (as of 11th March 2026). From these, 3572 and 4136 sequences were from unsegmented and segmented viruses, respectively. The number of segmented viruses, and thus of RdRp-encoding segments, was 1173, and the number of non-RdRp-encoding segments was 2963. We considered the set composed of the genomes of the unsegmented viruses and the RdRp-encoding segments of the segmented viruses as positive cases for this validation, which were 4745 sequences in total. The rationale for this validation analysis, and the Virushunter and Virusgatherer approach in general, is to identify RNA viruses by detecting their RdRp-encoding genomes or genome segments. Thus, we considered the set of 2963 non-RdRp-encoding genome segments of the segmented viruses as negative cases.
For each of the analyzed viral RefSeq sequences (positives and negatives) we generated an *in silico* dataset as follows. We randomly selected a desired average coverage depth of 2, 5, 10, 20, 50, or 100, reflecting the varying read coverage depths observed in real viral infections. We calculated the required number of reads, rnr, by multiplying the coverage depth by the viral reference sequence length and dividing by the read length (125), then rounding up to the next integer. We used InSilicoSeq (ISS) in basic mode to generate rnr read pairs from the input viral reference sequence. We added all reads from 1000 randomly selected eukaryotic mRNA sequences (see above) to the dataset.
All *in silico* generated datasets (n=7708, 580 GB in total) were analyzed using Virushunter and Virusgatherer in the same way as the main Chiroptera SRA data. Access to these data or a subset of it can be made available upon reasonable request.

Discrimination between RdRp-positive and RdRp-negative cases: We were unable to discriminate all RdRp-encoding from non-RdRp-encoding segments via the Genbank annotation metadata. To avoid a circular logic, we also did not detect the RdRp-containing segments by screening the viral RefSeq sequences with the RdRp pHMMs accompanying the Virushunter and Virusgatherer package. Instead, we took the following approach. We grouped the segment sequences by virus; this is possible because the virus annotation field of the RefSeq data has unique entries. For each virus with segmented genome, we considered the best hit (lowest E-value) to be the RdRp-encoding segment while all other segments were considered non-RdRp-encoding. Assuming a virus with K segments, this can result in the following scenarios: (1) exactly one hit is obtained, corresponding to 1 true positive and K-1 true negatives, (2) zero hits are obtained, corresponding to 1 false negative and K-1 true negatives, and (3) p hits are obtained with p>1, corresponding to 1 true positive, p-1 false positives, and K-p true negatives.

Sensitivity and specificity

We first assessed the end-to-end performance of the virus discovery workflow at the per-dataset level, involving both virus identification (Virushunter) and viral genome assembly (Virusgatherer). Virusgatherer performs a final protein-based BLAST analysis to annotate the assembled contigs. We considered a dataset to be a hit (virus identified) if at least one contig with a BLAST E-value of 10^{-5} or lower was found. If several contigs were assembled for an individual dataset, we used the smallest E-value across these contigs. We note that although the background eukaryotic mRNA sequences could also be considered to contribute to the negative cases, we disregarded them during the calculation of the performance measures, which therefore represent conservative estimates.

RdRp-encoding contigs were correctly assembled for 4566 of the 4745 positive-case datasets, corresponding to a sensitivity of 96.23% (**Additional Table 1)**. Of the 2963 negative cases, 2582 were correctly identified as not containing RdRp-encoding sequences, corresponding to a specificity of 87.14% (**Additional Table 1**). Overall, 7148 *in silico* datasets (4566 positives + 2582 negatives) were correctly classified, corresponding to an accuracy of 92.73% with a precision of 92.30%.

Additional Table 1. Assessment of sensitivity and specificity of the virus discovery workflow. 4745 *in silico* generated datasets with RdRp-encoding viral sequences (positives) and 2963 *in silico* generated datasets with non-RdRp-encoding viral sequences (negatives) were analyzed.

	positives	negatives	
identified	4566 (96.23 %)	381 (12.86 %)	
not identified	179 (3.77 %)	2582 (87.14 %)	
total	4745	2963	7708

We additionally assessed the spectrum of known RNA virus families that the workflow can identify using the set of profile HMMs included in the package. This corresponds to an assessment of only the Virushunter part of the workflow. Twenty-eight profile HMMs were considered and the deltavirus antigen profile was excluded from this validation analysis as deltaviruses belong to the separate realm *Ribozyviria*. We used the results of the profile HMM search in the *in silico* translated read data conducted by Virushunter and applied an hmmsearch E-value cut-off of 1 to call hits, requiring at least one viral read matching one or more of the RdRp profiles. Virushunter correctly identified viral reads in 4654 of the 4745 *in silico* datasets containing RdRp-encoding sequences, corresponding to a sensitivity of 98.08 %.
We then aggregated the hits at the virus family level by first calculating the smallest hmmsearch E-value across all reads identified for a particular dataset and then averaging these E-values across all datasets containing viral sequences from the same virus family. This was done for each profile HMM separately because a viral sequence may be detected by several HMMs. We then visualized these aggregated Virushunter search results as a heatmap in which rows represent the profile HMMs, columns represent the included RNA virus families and cells are colored according to the mean hmmsearch E-value (**Supplementary Figure 1**). Notably, each of the 119 virus families was detected by at least one of the profile HMMs. Most families were detected by several profile HMMs with a maximum of 18 for the family *Picornaviridae*. Each of the 28 profile HMMs detected several virus families, ranging between 2 and 50 for the Birnaviridae_RdRp and the Tombus-Noda_RdRp profile HMM, respectively (**Supplementary Figure 1**). These results demonstrated that Virushunter is capable of identifying the full spectrum of known RNA viruses.

Contig assembly completeness

Completeness of the virus-positive contigs assembled by Virusgatherer, calculated as the assembled contig length divided by the original sequence length of the viral sequence used for read simulation and multiplied by 100, varied considerably from 1 to 100 % (**Supplementary Figure 2**). The biggest increase in average assembly completeness was observed from simulated coverage depth 10 to 20; while only 3.7% of contigs simulated with coverage depth of 10 had a completeness of at least 90%, this value increased to 44.0% for coverage depth of 20. For simulated coverage depths of 50 and 100 the percentage of contigs with at least 90% completeness was 83.8 and 81.2, respectively.

Runtime and resource usage

We provide a summary of runtime and resource usage for the main analysis of Chiroptera SRA data conducted in this study. The size of analyzed SRA runs ranged from 1 MB to 13.03 GB, with an average size of 2.18 GB. For the Virushunter part of the workflow, the average runtime per GB per dataset was 90.5 minutes. For the Virusgatherer part of the workflow, the average runtime per GB per dataset was 110.6 minutes.

We ran the analyses on a high-performance computing (HPC) cluster. For each analyzed SRA dataset we used 12 CPUs and 50 GB of memory. We note that the actual elapsed real time (wall time) is strongly influenced by several parameters such as the hardware configuration of the cluster, cluster load, degree of parallelization, and more, thus, may not be readily transferable to other systems. The runtime of a dataset also depends on the amount of viral sequences present in the data due to the homology-based comparison of identified sequences against viral references.

Reference databases

Virushunter and Virusgatherer rely on several BLAST databases to filter and annotate assembled contigs. Each of them is specified in the config.yaml configuration file and can be substituted by user-provided databases. These databases need to be installed on the user’s system before running the pipeline. For more details, including installation instructions, see the documentation at the Virushunter and Virusgatherer GitHub repository.

The BLAST databases that we used in this study include:

- A custom contaminant database used to remove frequently occurring false-positive hits (included in the GitHub repository),
- The RefSeq protein database of NCBI used to remove remaining false-positive hits,
- The RefSeq viral genomic database of NCBI used to annotate true-positive hits.

Extended results

We have screened 3,348 publicly available transcriptome sequencing experiments from the SRA with Virushunter and Virusgatherer, resulting in 4,329 contigs of putative RNA virus origin. These contigs and their sequence similarity to the closest reference sequences from viral RefSeq protein are listed in **Supplementary Table S5**. The contigs were clustered at 90% sequence identity into 3,074 non-redundant sequences from 80 different RNA virus families. 33 of these contigs were longer than 5,000 nt and are shown in **Supplementary Figure S5**. The SRA run identifiers are part of the contig names, and the host taxa of the sequencing experiments are shown in **Supplementary Table S5**.

Five of these contigs are shown in **Figure 3F**. They include two coronavirus genomes from an *Otomops harrisoni* bat (contig SRR14579882_cap3_Contig-1 of length 26859 nt) and a *Molossus molossus* bat (SRR13259272_cap3_Contig-1, 19350 nt). Both genome sequences show the canonical ORF1a/b genomic architecture of coronaviruses and encode homologs of coronavirus non-structural proteins. The reconstructed coronavirus genome from the *M. molossus* bat is partial (only covering ORF1a/b) while the *O.harrisoni* virus is coding-complete and shows the 3’-proximal ORFs, including a predicted spike glycoprotein gene, as seen in other coronaviruses. The *M. molossus* virus was identified in a sample from Peru, and its genome shows an amino acid sequence identity of only ~70.63% to the closest reference virus (a bat alphacoronavirus), likely being the prototype of a new virus species within the genus *Alphacoronavirus*.

The third viral genome shown in **Figure 3F** is from a *Myotis lucifugus* bat (SRR6793287_cap3_Contig-1, 13467 nt) and shows the largest, but still remote, sequence similarity of 26.7% to an unclassified virga-like RNA virus (**Supplementary Table S5**). This virus likely represents a novel taxonomic group, and a proper taxonomic classification analysis is required to determine whether it is the prototype of a new virus family. The genome of this virus encodes a single, long ORF, resembling those of other RNA viruses.

The fourth sequence shown in **Figure 3F** is likely an endogenized element from a bornavirus that was integrated into the genome of a *Myotis daubentonii* bat (ERR13757851_cap3_Contig-1, 10244 nt). Only about half of the sequence encodes for ORFs longer than 300 nt, which would be highly unusual for a genome of an exogenous RNA virus. The contig shows 58.4% sequence identity to a fish (Wuhan sharpbelly) bornavirus, suggesting that the viral genome, which supposedly integrated into the bat genome, was from an unknown bornavirus species. Related, Wuhan sharpbelly bornavirus-like sequences were identified in 94 sequencing experiments from 22 different projects (**Supplementary Table S5**). These studies analyzed data from 13 different bat species of 7 different genera, suggesting that the putative bornavirus integration happened more than 50 million years ago in the ancestor of these bats (according to

the divergence times provided by TimeTree.org for the 13 bat species) or that several integration events of very similar bornaviruses happened during bat genome evolution. Notwithstanding that, the identification of these bornavirus-like sequences demonstrates that Virushunter and Virusgatherer can also be used for identifying endogenous viral elements besides its original purpose of discovering exogenous viral genomes.

The fifth viral genome shown in **Figure 3F** is from a *Barbastella barbastella* bat (SRR28553504_cap3_Contig-1, 10242 nt) and shows the largest sequence similarity of 46.7% to an unclassified rhabdovirus (Agrotis ipsilon virus) (**Supplementary Table S5**). This viral genome shows the canonical multi-ORF architecture of rhabdoviruses with an L-like gene encoding the viral polymerase at the 5'-proximal part of the genome. This virus likely is the prototype of a novel virus species and may also represent a new genus in the family *Rhabdoviridae*.

Supplementary references

1. Shi,M., Lin,X.-D., Tian,J.-H., Chen,L.-J., Chen,X., Li,C.-X., Qin,X.-C., Li,J., Cao,J.-P., Eden,J.-S., *et al.* (2016) Redefining the invertebrate RNA virosphere. *Nature*, **540**, 539–543.

2. Schulz,F., Roux,S., Paez-Espino,D., Jungbluth,S., Walsh,D.A., Denef,V.J., McMahon,K.D., Konstantinidis,K.T., Eloe-Fadrosh,E.A., Kyrpides,N.C., *et al.* (2020) Giant virus diversity and host interactions through global metagenomics. *Nature*, **578**, 432–436.

3. Krupovic,M., Varsani,A., Kazlauskas,D., Breitbart,M., Delwart,E., Rosario,K., Yutin,N., Wolf,Y.I., Harrach,B., Zerbini,F.M., *et al.* (2020) Cressdnaviricota: a Virus Phylum Unifying Seven Families of Rep-Encoding Viruses with Single-Stranded, Circular DNA Genomes. *J Virol*, **94**, e00582-20.

4. Lauber,C., Seitz,S., Mattei,S., Suh,A., Beck,J., Herstein,J., Börold,J., Salzburger,W., Kaderali,L., Briggs,J.A.G., *et al.* (2017) Deciphering the Origin and Evolution of Hepatitis B Viruses by Means of a Family of Non-enveloped Fish Viruses. *Cell Host Microbe*, **22**, 387-399.e6.

5. Gourlé,H., Karlsson-Lindsjö,O., Hayer,J. and Bongcam-Rudloff,E. (2019) Simulating Illumina metagenomic data with InSilicoSeq. *Bioinformatics*, **35**, 521–522.

Supplementary Figures

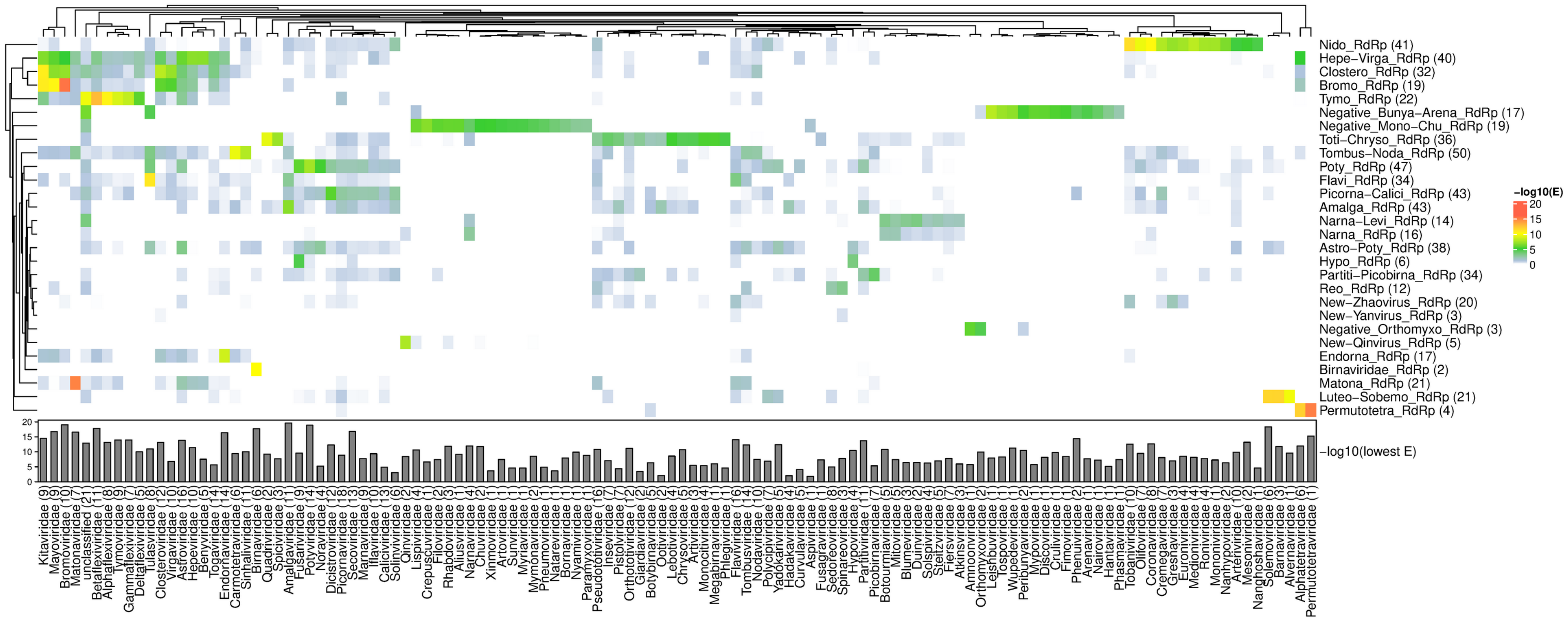

Supplementary Figure S1. Virushunter can detect the full spectrum of known RNA viruses. The heatmap shows Virushunter's sensitivity of detecting RNA virus sequences in 4745 *in silico*-generated sequencing datasets containing reads from RdRp-encoding sequences of 119 different RNA virus families (columns). Each dataset was screened using 28 RdRp profile HMMs (rows). Hmmsearch E-values were averaged across all datasets that contained viral sequences from the same virus family. Heatmap cell colors reflect these average E-values. The number in brackets following a virus family name (x-axis) indicates how many profile HMMs gave significant hits (E < 1) against that family, while the number in brackets following a profile HMM name (y-axis) shows the number of virus families against which this profile HMM gave significant hits. The bar chart below the heatmap shows the lowest E-value (in -$\log_{10}$ scale) across all profile HMMs for each virus family without averaging across datasets.

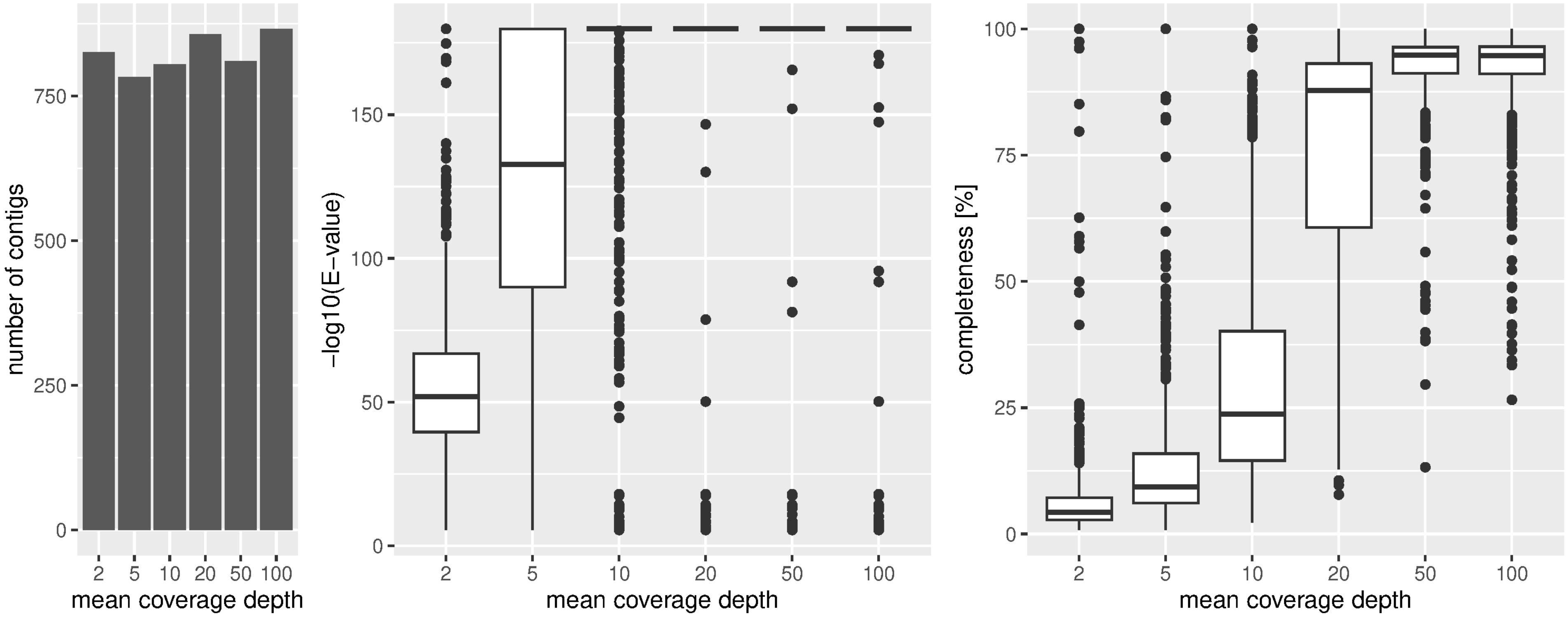

Supplementary Figure S2. Assembly statistics for the virus-positive contigs reconstructed by Virusgatherer from the *in silico*-generated sequencing datasets for the different mean coverage depth values used in the simulation. Left: number of assembled contigs. Middle: best E-value in the comparison of each assembled contig against the viral RefSeq database. Right: Assembly completeness as the percentage of the original sequence length covered by the assembled sequence.

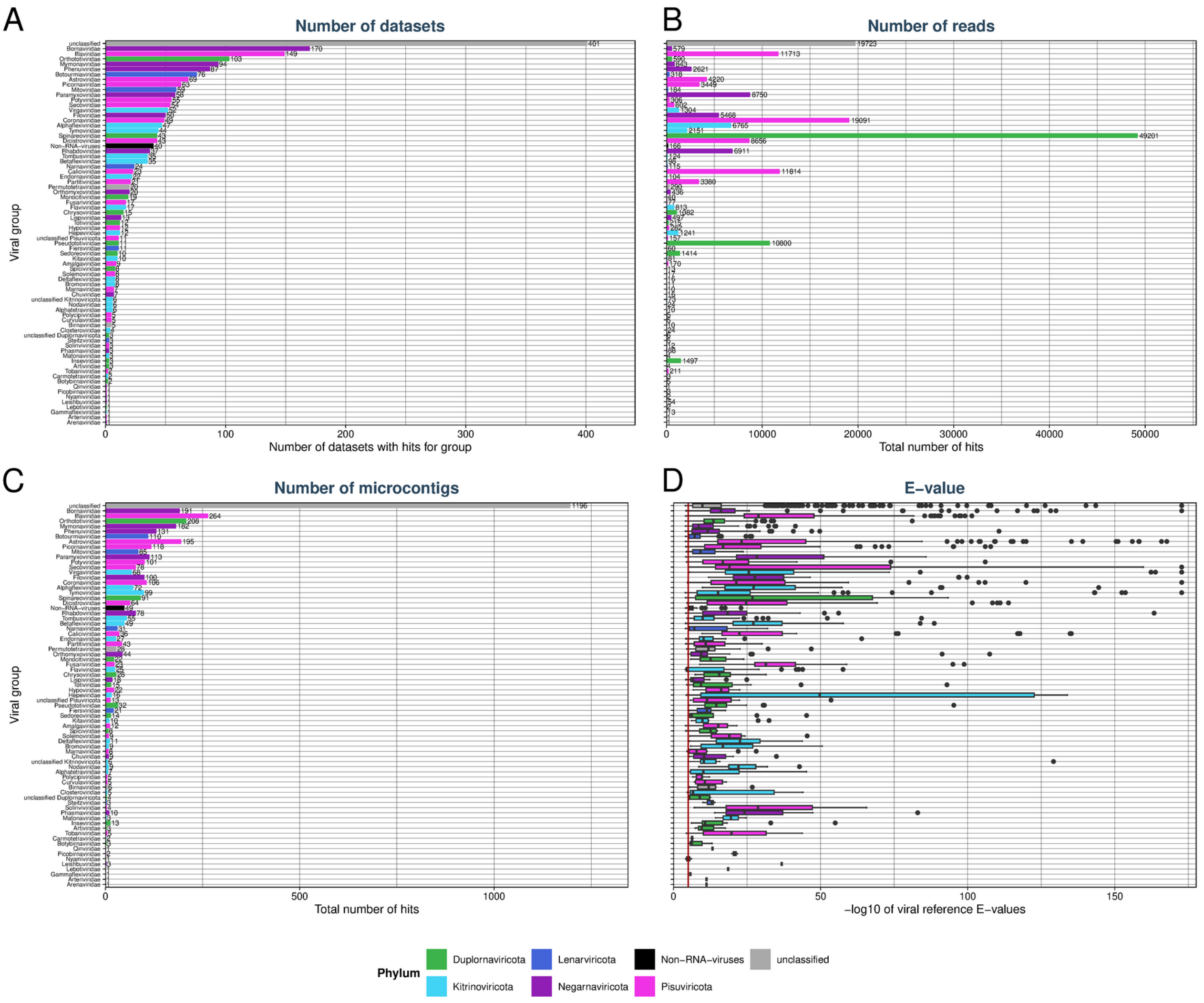

Supplementary Figure S3. Full Virushunter results of the main SRA-based analysis grouped by virus family and colored by virus phylum. (A) Number of identified SRA datasets, (B) number of identified sequencing reads, (C) number of microcontigs, (D) distribution of E-values of protein Blast search against RNA virus reference sequences.

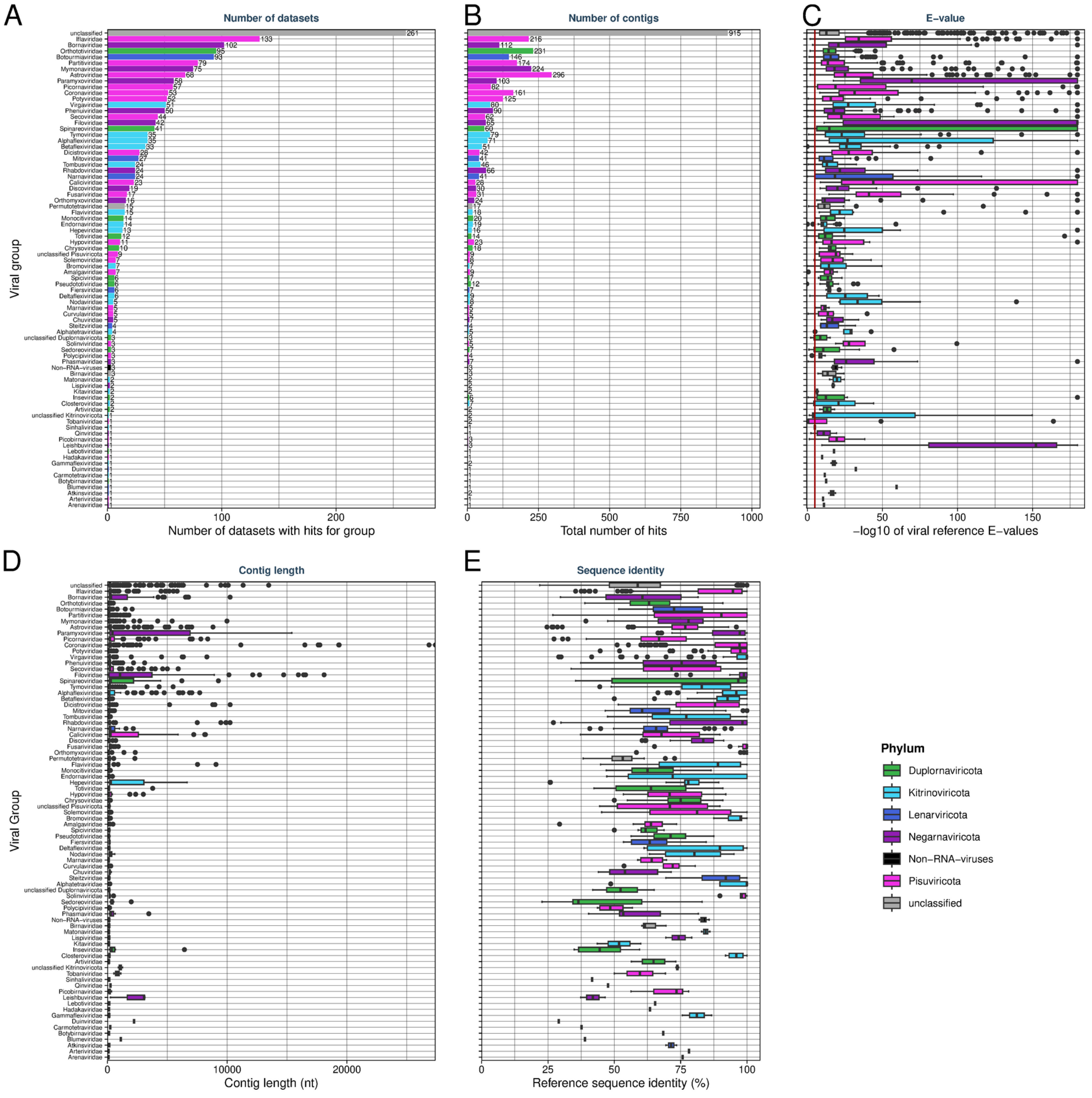

Supplementary Figure S4. Full Virusgatherer results of the main SRA-based analysis grouped by virus family and colored by virus phylum. (A) Number of identified SRA datasets, (B) number of contigs, (C) distribution of E-values of protein Blast search against RNA virus reference sequences, (D) distribution of contig lengths, (E) distribution of protein sequence identity to closest reference.

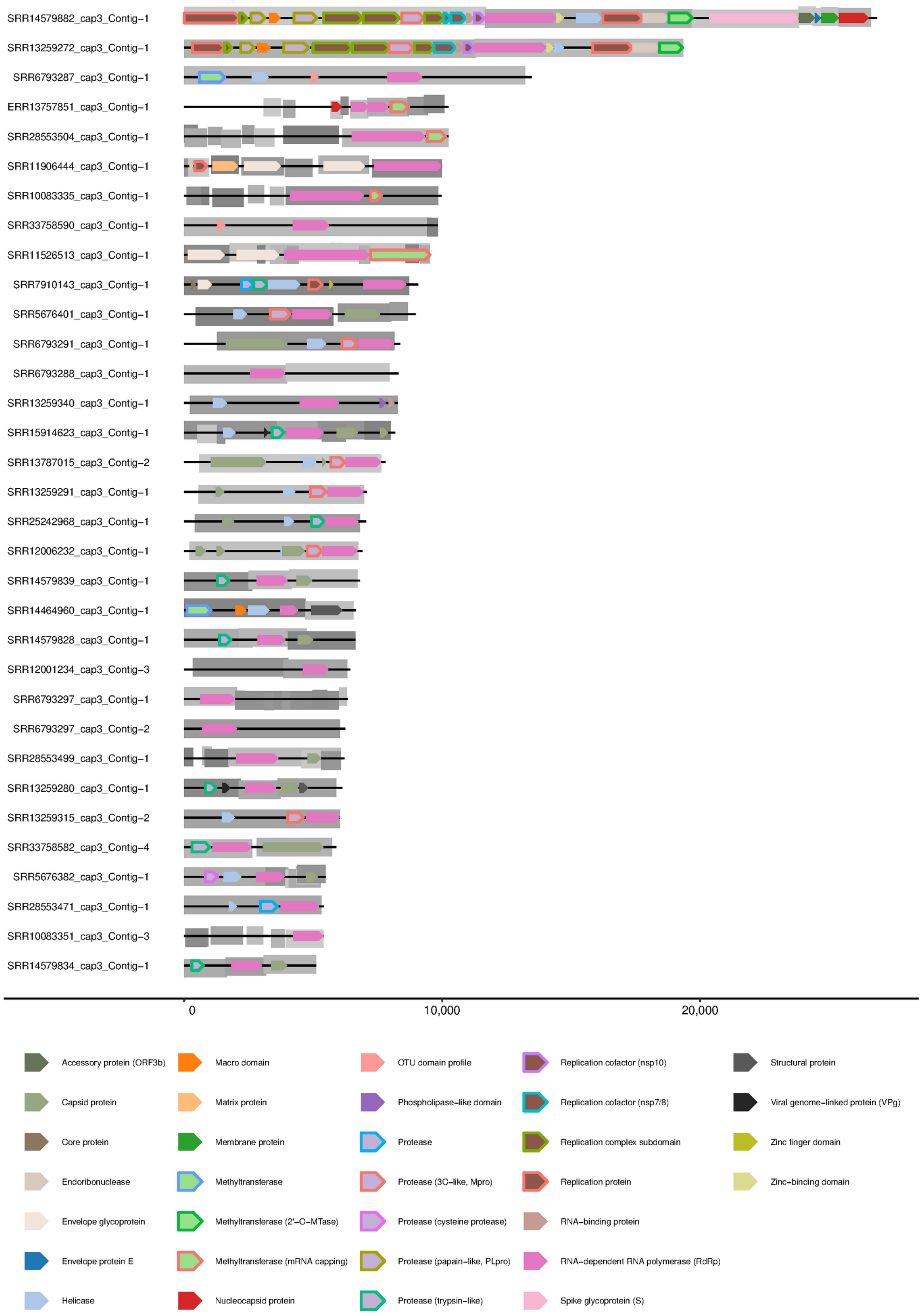

Supplementary Figure S5. Genome maps of 33 discovered non-redundant RNA virus genomes of at least 5000 nt and amino acid sequence identity to viral RefSeq representatives of less than 80%. Predicted open reading frames (ORFs) of at least 300 nt are shown as rectangles with different shadings of gray. Protein domains predicted in an InterProScan analysis are shown as colored shapes within ORFs; see color legend at the bottom. For detailed virus annotations, see Supplementary Table S6.
